# Capsule deletion via a λ-Red knockout system perturbs biofilm formation and fimbriae expression in *Klebsiella pneumoniae* MGH 78578

**DOI:** 10.1186/1756-0500-7-13

**Published:** 2014-01-08

**Authors:** Tzu-Wen Huang, Irene Lam, Hwan-You Chang, Shih-Feng Tsai, Bernhard O Palsson, Pep Charusanti

**Affiliations:** 1 Institute of Molecular and Genomic Medicine, National Health Research Institutes, 350 Zhunan, Taiwan; 2 Department of Bioengineering, University of California, San Diego La Jolla, CA, 92093-0412 USA; 3 Institute of Molecular Medicine, National Tsing Hua University, 30013 Hsinchu, Taiwan

**Keywords:** Klebsiella pneumoniae, Capsule, Biofilm, Fimbriae, Expression profiling, Gene knockouts, Transmission electron microscopy, Infectious disease

## Abstract

**Background:**

*Klebsiella pneumoniae* is a leading cause of hospital-acquired urinary tract infections and pneumonia worldwide, and is responsible for many cases of pyogenic liver abscess among diabetic patients in Asia. A defining characteristic of this pathogen is the presence of a thick, exterior capsule that has been reported to play a role in biofilm formation and to protect the organism from threats such antibiotics and host immune challenge.

**Findings:**

We constructed two knockout mutants of *K. pneumoniae* to investigate how perturbations to capsule biosynthesis alter the cellular phenotype. In the first mutant, we deleted the entire gene cluster responsible for biosynthesis of the extracellular polysaccharide capsule. In the second mutant, we deleted the capsule export subsystem within this cluster. We find that both knockout mutants have lower amounts of capsule but produce greater amounts of biofilm. Moreover, one of the two mutants abolishes fimbriae expression as well.

**Conclusions:**

These results are expected to provide insight into the interaction between capsule biosynthesis, biofilm formation, and fimbriae expression in this organism.

## Findings

### Introduction

*Klebsiella pneumoniae* is a Gram-negative bacterium that is a member of the family Enterobacteriaceae and is closely related phylogenetically to the genera *Escherichia*, *Salmonella*, *Shigella*, and *Yersinia*. Medically, *K. pneumoniae* causes a wide range of diseases worldwide such pneumonia, urinary tract infections, and surgical wound infections that primarily afflict immunocompromised patients in hospital settings and long-term care facilities. The number of community-acquired infections caused by *K. pneumoniae*, however, has increased worldwide over the past several decades. For example, invasive forms of the disease characterized by bacteremic liver abscesses or endophthalmitis that are contracted in the community are endemic in Asia [1-3], especially in Taiwan [4-7], and reports of their occurrence are now emerging in other parts of the world [8,9]. Infections caused by *K. pneumoniae* can be difficult to treat since many clinical isolates possess an extensive repertoire of antibiotic resistance genes. Ominously, some strains have now been isolated from different parts of the world that harbor New Delhi metallo-β-lactamase 1 (NDM-1) [10,11], a gene that confers resistance to carbapenem antibiotics, the last-line treatment option against most *K. pneumoniae* infections. Compounding the medical threat is the paucity of new antibiotics that are being developed against multi-drug resistant Gram-negative bacteria such as *K. pneumoniae*.

Despite its close relationship to other enterobacteria, one notable difference between *K. pneumoniae* and other members of this family is the presence of an extremely thick, hypermucoviscous, extracellular polysaccharide capsule that surrounds this bacterium*.* At least 77 distinct capsular serotypes have been reported to date [12], but virulent strains have been predominantly associated with the K1 and K2 serotypes, in particular K1 [12-15]. The genes responsible for biosynthesis of the capsule are normally located in a cluster that is 21 to 30 kb in length and comprise 16 to 25 ORFs [16]. The capsule is believed to be a major virulence determinant by protecting *K. pneumoniae* from phagocytosis [17-19] and destruction by antimicrobial peptides [20]. Furthermore, the capsule is thought to play a crucial role in biofilm formation, which allows the organism to colonize indwelling medical devices and better survive hostile conditions such as detergents aimed at removing the biofilms, since mutants of *K. pneumoniae* strain LM21 with disruptions in different genes involved in capsule biosynthesis produce less biofilm [21]. Consistent with these findings, a separate signature-tagged mutagenesis study also identified a mutation in ORF12 of the *K. pneumoniae* strain 43816 K2 capsule gene cluster that resulted in less biofilm formation [22]. On the other hand, a third study found that a non-encapsulated derivative of *K. pneumoniae* strain C105 produced greater amounts of biofilm than the parental strain, and correlated this observation to the expression of type 1 fimbriae [23]. A fourth study found that type 3 fimbriae promoted biofilm formation in a strain that still possessed its capsule [24].

These findings in aggregate highlight the complex interaction between capsule biosynthesis, biofilm formation, and fimbriae expression. Against this backdrop, we investigated the relationship among encapsulation, biofilm formation, and fimbriae expression in *K. pneumoniae* strain MGH 78578. Significantly, we found that a non-encapsulated mutant produced larger amounts of biofilm as has been reported [23], but in contrast also found that no fimbriae was required for this phenotype.

### Materials and methods

#### Strains and primers

*Klebsiella pneumoniae* strain MGH 78578 was purchased from ATCC. All other strains were generated as part of this study (Table 1). All primers for plasmid construction and gene knockouts can be found in Additional file 1.

**Table 1:** List of strains and plasmids used in this study

**Strains/Plasmids**	**Description**	**Source**
***K. pneumoniae strains***		
MGH 78578	Clinical isolate from a pneumonia patient. Parental (wild-type) strain for gene deletion.	ATCC
∆wzabc	In-frame deletion of wzabc operon (KPN_02510-KPN_02512)	This study
∆cps	In-frame deletion of capsule biosynthesis cluster ranging from ugd (KPN_02493) to galF (KPN_02515)	This study
***E. coli strains***		
Top10	Competent cells for general cloning	Life Technologies
**Plasmids**		
pACBSR	A p15A replicon plasmid containing an arabinose-inducible λ-Red recombinase and chloramphenicol resistance selection marker	[25]
pEXP5-CT	General vector containing ampicillin resistance used during sub-cloning to make pACBSR-Hyg	Life Technologies
pSecTag/FRT/V5-His	Vector containing a promoter-less hygromycin resistance gene	Life Technologies
pEXP5-CT-hyg	Same as pEXP5-CT, but with the ampicillin resistance marker replaced with a hygromycin resistance marker	This study
pCP20	Plasmid bearing a heat-shock inducible FLP recombinase	[26]
pIJ773	Template for amplification of the apramycin resistance cassette	[27]
pACBSR-Hyg	Same as pACBSR, but with the chloramphenicol resistance marker replaced with a hygromycin resistance marker	This study
pFLP-Hyg	Same as pCP20, but with the pSC101 replicon and chloramphenicol resistance marker of pCP20 replaced with the p15A replicon and hygromycin resistance marker from pACBSR-Hyg	This study

#### Growth media

All experiments were conducted using bacteria grown in LB, low salt LB, or glucose M9 media. Low salt LB consisted of (per liter) 5 g yeast extract, 10 g tryptone, and 5 g NaCl, and was adjusted to pH 8.0 with NaOH. Glucose M9 was composed of the following chemicals (per liter): 6.8 g Na_2_HPO_4_; 3 g KH_2_PO_4_; 0.5 g NaCl; 1 g NH_4_Cl; 100 μM CaCl_2_; 2 mM MgSO_4_; 2 g dextrose. Stock solutions of CaCl_2_, MgSO_4_, and dextrose were filter sterilized through Millipore ExpressPlus 0.22 μm membranes (Millipore, Billerica, MA) and subsequently added individually to autoclaved solutions containing the first four chemicals. Hygromycin (Sigma-Aldrich, St. Louis, MO) and apramycin (Research Products International, Mt. Prospect, IL) were added to the media to final concentrations of 100 μg/ml and 50 μg/ml, respectively, as necessary. Low salt LB and hygromycin are always used together; all other steps use standard LB.

#### Construction of knockout mutants

A detailed description of the knockout procedure and plasmid maps can be found in Additional file 2. A similar protocol was recently published [28].

#### Capsule quantification

The capsule surrounding *K. pneumoniae* MGH 78578 belongs to the K52 serotype, which has a hexasaccharide repeating unit composed of two rhamnose, one glucose, one glucuronic acid, and two galactose sugars [29]. Measurements of the amount of capsule surrounding wild-type *K. pneumoniae* MGH 78578 and all the mutants were based on the protocol of Domenico, *et al.*[30].

#### Transcriptome profiling

For transcriptome profiling, a high-density oligonucleotide tiling array consisting of 379,528 50-mer probes spaced 30 bp apart across the whole *K. pneumoniae* MGH 78578 genome was custom-designed by NimbleGen (Roche). Total RNA from OD_600_ ~0.5 cultures were hybridized to the arrays according to the protocol of Qiu, et al. [31]. The normalized probe level information was transformed into expression level data for each gene using the Genbank annotation for *K. pneumoniae* MGH 78578 (accession number PRJNA57619). Genes were deemed differentially expressed between the two mutants and the wild-type if there was a 2-fold or greater change and they had a p-value less than 0.05. The expression profiling datasets of *K. pneumoniae* wild-type and capsule deletion mutant (Δcps) have been deposited in the Gene Expression Omnibus (GEO) database and assigned the accession number GSE40011. Three biological replicates of the wild-type, Δcps, and Δwzabc strains were used to generate the array data.

#### Transmission electron microscopy imaging

The samples were negatively stained with 1% aqueous uranyl acetate and examined on an FEI Tecnai G2 Sphera transmission electron microscope at 200 keV. Images were recorded on a Gatan Ultrascan UHS CCD camera.

### Results and discussion

#### Deletion of the entire capsule biosynthesis cluster and the export subsystem leads to phenotypic defects in the two mutants

We developed a gene knockout procedure for *K. pneumoniae* that is based on the widely-used *E. coli* λ-Red recombinase system. The independent development of a similar protocol was recently reported [28]. The method developed here requires two selection markers, one (apramycin resistance) is used to replace the target gene and the other (hygromycin resistance) selects for two plasmids used at different times to mediate homologous recombination and excision of the apramycin resistance cassette.

We examined how different homology lengths affect transformation efficiency by deleting the same locus in *K. pneumoniae* using cassettes that contained 39-bp, 60-bp, and 700-bp homology to the target region. This target region was the three genes (*wza*, *wzb*, and *wzc*) that function in capsule export. A total of one microgram of each of the three PCR products was electroporated into the host. We recovered 26, 46, and 255 apramycin-resistant transformants using the 39-bp, 60-bp, and 700-bp homology lengths, resulting in transformation efficiencies of 1.3 × 10^-8^, 2.5 × 10^-8^, and 1.1 × 10^-7^, respectively. To confirm these mutant candidates, we next examined 24 colonies from each group by PCR. None of the 24 colonies from the 39-bp group was a correct knockout mutant, whereas two colonies from the 60-bp group and ten colonies from the 700-bp group were correct (data not shown). Although longer homology lengths resulted in higher transformation efficiency, these results implied that replacement cassettes containing 60-bp homology were sufficient to create knockout mutants. The construction of such cassettes requires DNA oligos that are only 80–85 nucleotides long, which can be readily purchased from commercial vendors. The use of knockout cassettes bearing longer homology arms would require an additional cloning step. For these reasons, we constructed all knockout mutants in this study using 60-bp homology.

**Figure 1:**
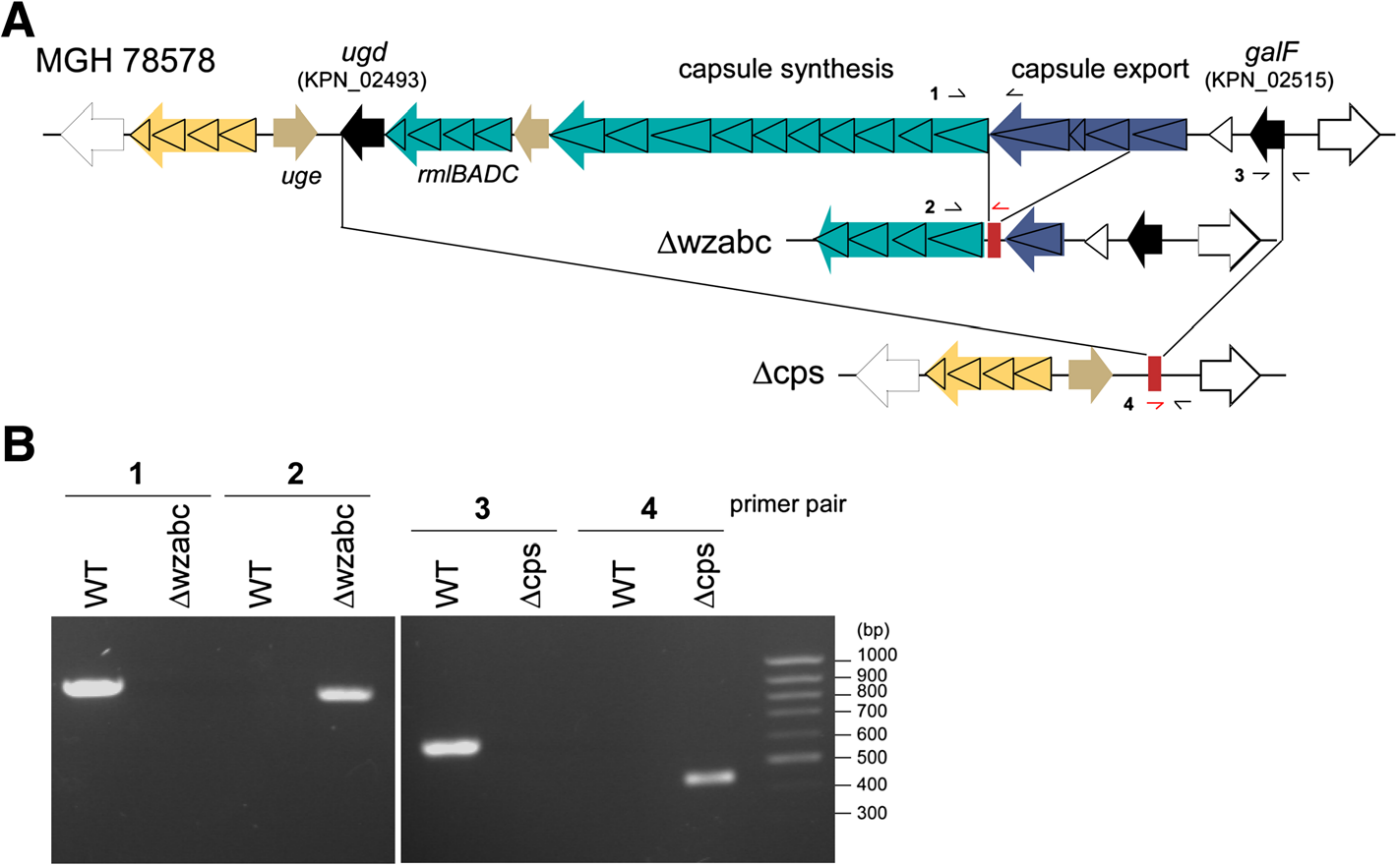
**Construction of two capsule defective mutants, Δcps and Δwzabc. A**. Genetic loci that were deleted in the two mutants ∆wzabc and ∆cps. A portion of the capsule export system within the capsule biosynthesis cluster was deleted in ∆wzabc whereas the entire biosynthesis cluster was deleted in ∆cps. The deletions in both mutants left behind an 81 bp scar region (red box). The triangles indicate the CDSs inside the gene clusters. **B**. Gel image confirming the deletions in both mutants. Two primer pairs (1 and 2 for ∆wzabc; 3 and 4 for ∆cps) were used to verify the deletions by PCR. The small arrows in part A denote the approximate binding location of the primers used to generate these amplicons. Pairs 1 and 3 were designed to amplify the 5′ and 3′ junctions of the wild-type sequence, respectively, while pairs 2 and 4 were designed such that either the forward or reverse primer from each pair bound inside the scar region. In this way, pairs 1 and 3 will produce a PCR amplicon if the wild-type sequence is still present, whereas pairs 2 and 4 amplify only if the correct target locus has been deleted. The amplicon sizes are: pair 1, 833 bp; pair 2, 741 bp; pair 3; 602 bp; pair 4, 428 bp.

We deployed our knockout protocol to investigate how the deletion of genes involved in biosynthesis of the thick, extracellular polysaccharide capsule surrounding *K. pneumoniae* perturbs the bacterium. We constructed two knockout mutants (Figure 1): the entire capsule biosynthesis cluster was deleted in one mutant, Δcps, whereas three genes responsible for capsule export (the wza-b-c operon) were deleted in the other mutant, Δwzabc (Table 1) [32,33]. Both deletions are marker-free, and we confirmed that the wild-type allele was completely absent in the two mutants by PCR and Sanger sequencing (Figure 1B).

**Figure 2:**
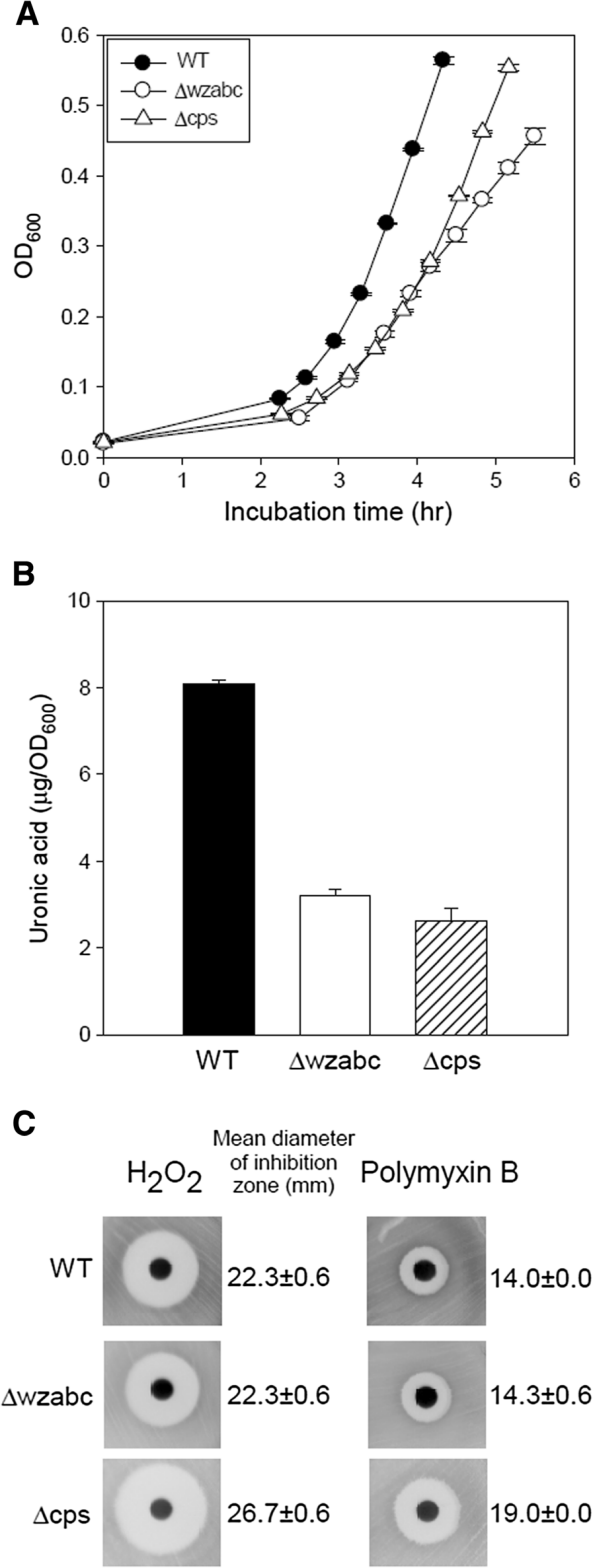
**Phenotypic characterization of both capsule deletion mutants. A**. Growth curves for the wild-type (WT), ∆wzabc, and ∆cps strains. **B**. Measurements of uronic acid content in the wild-type, ∆wzabc, and ∆cps strains. Values were normalized by the optical densities of the samples when the measurements were made. **C**. The susceptibility of the wild-type, ∆wzabc, and ∆cps strains to hydrogen peroxide (left) and polymyxin B (right). Error bars in all three panels denote the standard deviation of three biological replicates.

The growth rates of the wild-type and Δcps strains during batch culture in glucose M9 were 1.022 ± 0.0028 and 0.808 ± 0.0057 (1/hour), respectively, indicating a statistically significant decrease in the growth rate (*P* < 5 × 10^-6^, Student’s t-test) (Figure 2A). Growth rates were calculated from time-course OD_600_ measurements. The Δwzabc mutant showed a biphasic growth pattern in which the growth rate during the first four hours was identical to that of the wild-type (1.026 ± 0.048 1/hour) but dropped to 0.49 ± 0.010 (1/hour) thereafter (Figure 2A).

The quantity of uronic acid that can be extracted from cells provides an indirect measure of the amount of extracellular capsule [34-37]. When compared to wild-type levels, the quantity of uronic acid in the Δcps and Δwzabc mutants declined to 0.29 ± 0.17 and 0.38 ± 0.04 fold change, respectively (*P* < 7.5 × 10^-4^ for both, Student’s t-test) (Figure 2B). As a negative control, we performed the same assay side-by-side using blank media only. Absorbance measurements for these control samples were consistently below the detection threshold of our spectrophotometer.

We next measured the sensitivity of the wild-type, Δcps, and Δwzabc strains to oxidative stress and the antimicrobial peptide polymyxin B since the absence of capsule was expected to increase sensitivity to these two stresses. Both measurements were carried out using a disc diffusion assay in which hydrogen peroxide or polymyxin B was impregnated into the disc and then placed onto agar plates covered by a lawn of each strain. The diameter of the inhibition zone of the wild-type and Δwzabc mutant to hydrogen peroxide was 22.3 mm in contrast to 26.7 mm for the Δcps mutant (*P* < 0.001, Student’s t-test) (Figure 2C). Similarly, the antimicrobial peptide polymyxin B assay produced a diameter of 19.0 mm each time when three independent tests on Δcps were performed, but only 14 mm on wild-type and Δwzabc (Figure 2C).

#### Expression profiling of the Δcps and Δwzabc deletion mutants reveals interplay between capsule biosynthesis and phenotypic defects

The phenotypic changes we observed in the Δcps capsule deletion mutant motivated us to examine gene expression differences between the Δcps and Δwzabc mutants versus the wild-type strain. Of 112 genes up-regulated more than two-fold (Table 2), those with the greatest fold increase belonged to the gene cluster KPN_04512 – KPN_04515, which is annotated as the *pga* operon that is responsible for synthesis of poly-beta-1,6-N-acetyl-D-glucosamine (PGA or poly-GlcNAc), a secreted, extracellular component of the biofilm matrix [38]. The transcription level of the *pga* operon was also slightly elevated in the Δwzabc mutant (Additional file 3).

**Table 2:** **List of the 20 genes most significantly up- and down-regulated in the** Δ**cps mutant relative to wild-type levels**

**Locus**	**Gene product**	**Expression level (wild-type)**	**Expression level (**Δ**cps)**	***p*-value**	**Fold change**
**Up-regulated genes**					
KPN_00042	Hypothetical	9.00	13.38	0.0021	20.82
KPN_00321	Hypothetical	6.63	9.20	0.0007	5.92
KPN_01030	Hypothetical	8.15	10.65	0.0170	5.66
KPN_01107	Hypothetical	9.41	13.32	0.0021	15.03
KPN_01226	Periplasmic protein	10.59	13.27	0.0052	6.38
KPN_01279	Lipoprotein, osmotically inducible	11.03	13.96	0.0012	7.62
KPN_01568	Hypothetical	7.46	10.07	0.0085	6.10
KPN_01977	Lysozyme inhibitor	9.57	12.45	0.0007	7.34
KPN_02742	Hypothetical	8.95	11.38	0.0057	5.40
KPN_03160	Hypothetical	11.17	13.82	0.0028	6.30
KPN_04221	Periplasmic repressor CpxP	10.08	12.49	0.0044	5.32
KPN_04433	Putative stress-response protein	9.46	11.81	0.0083	5.09
KPN_04512^*a*^	N-glycosyl-transferase PgaC	6.40	9.74	0.0003	10.13
KPN_04513^*a*^	Putative polysaccharide deacetylase	6.38	9.79	0.0006	10.67
KPN_04514^*a*^	Outer membrane protein PgaA	6.43	10.95	0.0002	22.95
KPN_04515^*a*^	Hypothetical	6.56	12.30	0.0059	53.44
KPN_04516^*a*^	Hypothetical	7.64	11.25	0.0010	12.21
KPN_04684	Putative sulfate transporter	8.78	11.67	0.0013	7.41
KPN_04685	Putative carbonic anhydrase	10.50	13.32	0.0049	7.02
KPN_04773	Putative porin	9.17	12.04	0.0026	7.31
**Down-regulated genes**					
KPN_00464	Copper exporting ATPase	10.44	8.48	0.0024	0.26
KPN_00626	methylthioribose kinase MtnK	11.02	9.38	0.0052	0.32
KPN_00688	tRNA-Gln-CTG	13.17	11.34	0.0213	0.28
KPN_00689	tRNA-Gln-CTG	13.01	11.26	0.0287	0.30
KPN_01055	tRNA-Ser-GGA	13.00	11.11	0.0341	0.27
KPN_01427	Hypothetical	9.12	7.35	0.0040	0.29
KPN_02433	tRNA-Asn-GTT	15.01	12.52	0.0365	0.18
KPN_02448	tRNA-Asn-GTT	14.91	12.54	0.0246	0.19
KPN_02488	dTDP-4-dehydrorhamnose 3,5-epimerase	12.69	11.02	0.0032	0.31
KPN_02489	dTDP-4-dehydrorhamnose reductase	13.06	11.18	0.0035	0.27
KPN_02593	Colicin I receptor	14.38	12.83	0.0034	0.34
KPN_02746	tRNA-Ala-GGC	10.11	8.52	0.0257	0.33
KPN_02747	tRNA-Ala-GGC	10.36	8.87	0.0377	0.36
KPN_02920	Bifunctional chorismate mutase/prephenate dehydratase	11.74	10.24	0.0092	0.35
KPN_03277^*b*^	Putative fimbrial usher protein	13.08	11.49	0.0024	0.33
KPN_03278^*b*^	Putative pili assembly chaperone	13.66	12.05	0.0024	0.33
KPN_04263	tRNA-Trp-CCA	10.89	8.38	0.0088	0.18
KPN_04300	tRNA-Pro-TGG	9.93	8.11	0.0193	0.28
KPN_04425	Maltoporin	11.34	9.61	0.0051	0.30
KPN_04776	Carbon starvation protein	9.26	7.63	0.0043	0.32

^*a*^Genes associated with the *pga* operon.

^*b*^Genes associated with the *mrk* type 3 fimbriae operon.

Eighty-nine genes were down-regulated more than two-fold in the Δcps mutant and fell within several groups: 17 plasmid-borne genes from pKPN3 and pKPN4, 13 metabolic genes, 11 tRNA genes, and eight genes associated with type 1 and type 3 fimbriae. Of the eight fimbriae-associated genes, five were located within the same cluster (KPN_03274 – KPN_03278) (Additional file 3). Genbank currently does not associate this set of genes with a specific type of fimbriae; however, each one is 100% homologous at the amino acid level to the *mrk*JFDCB cluster from *K. pneumoniae* NTUH-2044 that encodes type 3 fimbriae. We therefore assume that this set of five genes encodes type 3 fimbriae in *K. pneumoniae* MGH 78578 as well. The other three (*fimA*, *fimI*, and *fimC*; KPN_03287 – KPN_03289) (Additional file 3) are located within a cluster whose gene product is annotated to be type 1 fimbriae.

#### Fimbriae biosynthesis is abolished in Δcps but not Δwzabc

**Figure 3:**
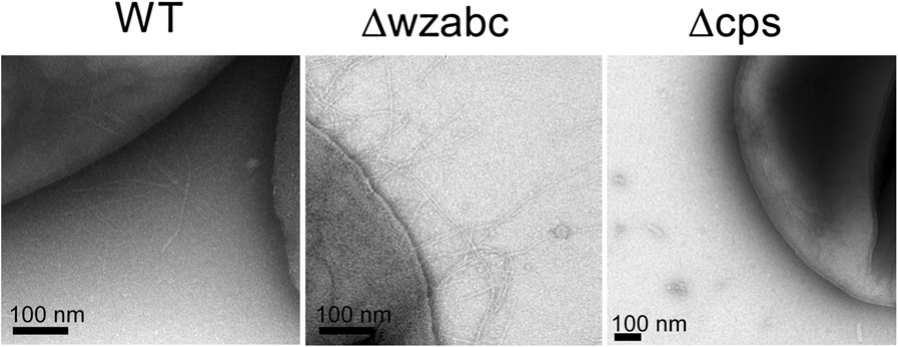
**Transmission electron microscopy images of wild-type *K. pneumoniae *and the** Δ**wzabc and** Δ**cps mutants.** The wild-type and ∆wzabc are fimbriated, whereas ∆cps is not. All images were taken at 19,000X nominal magnification (27,290X CCD magnification).

In lieu of standard RT-PCR, we interrogated the findings from expression analysis through a series of corresponding phenotypic assays. The down-regulation of genes encoding both type 1 (*fim* cluster) and type 3 (*mrk* cluster) fimbriae raised the possibility that fimbriation had declined in the two mutants. To investigate this possibility, we visualized the surface structure of the wild-type and the two knockout mutants using transmission electron microscopy (TEM). Fimbriae could be detected on the surface of the wild-type and Δwzabc mutant but not on the Δcps mutant (Figure 3).

#### Up-regulation of the pga operon appears to be a more important factor in biofilm formation than down-regulation of the fimbriae-associated operons

**Figure 4:**
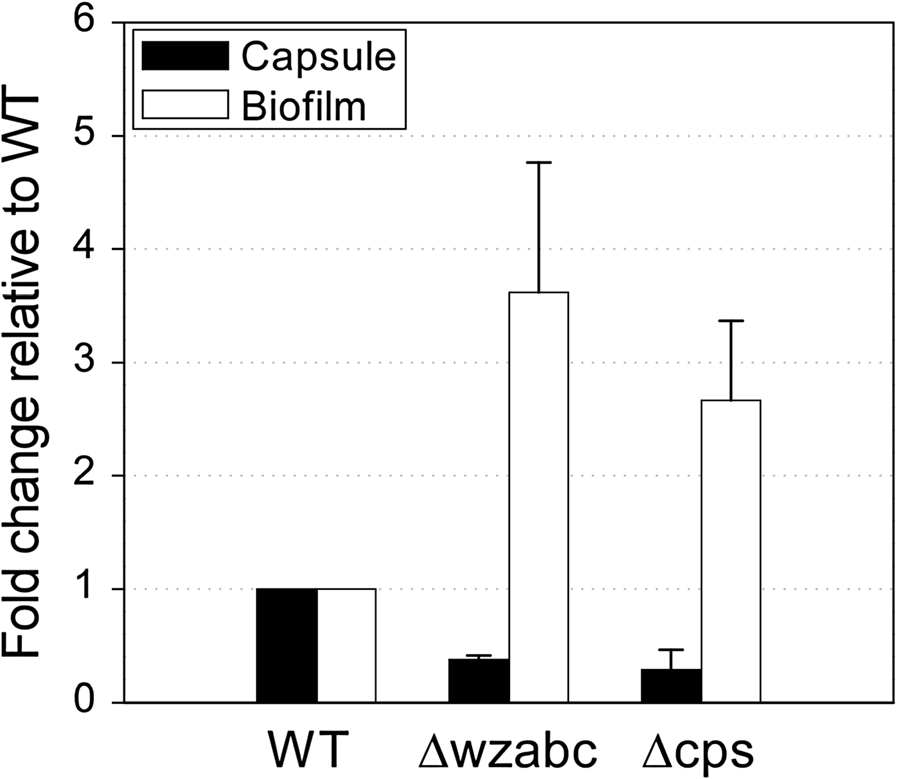
**The fold change in the amount of uronic acid (capsule) and biofilm produced by the** Δ**cps and** Δ**wzabc mutants relative to the wild-type (WT).** Error bars represent the standard deviation of three biological replicates. The uronic acid data is identical to that found in Figure 2 but presented here as fold change.

Type 1 fimbriae has been previously linked to increased biofilm formation in a non-encapsulated *K. pneumoniae* mutant [23]. In our non-encapsulated Δcps mutant, however, we observed twice as much biofilm relative to wild-type levels (Figure 4) but no fimbriae (Figure 3) when using a crystal violet assay to quantify the amount of biofilm [39]. These observations suggest that upregulation of the *pga* operon alone is sufficient to promote biofilm formation in non-encapsulated mutants; fimbriae are not needed. The Δwzabc mutant produced even greater amounts of biofilm (four-fold increase) when compared to the wild-type (Figure 4), but the *pga* operon was overexpressed only 1.7-fold (Additional file 3). The continued presence of fimbriae suggests that, in this mutant, both the *pga* operon and fimbriae probably act synergistically to promote biofilm.

### Concluding remarks

In this study, we observed a complex interplay among encapsulation, biofilm formation, and fimbriae expression in *Klebsiella pneumoniae* MGH 78578. This strain exhibited increased biofilm formation despite the absence of capsule, an effect likely stemming from strong up-regulation of the *pga* operon. In contrast, other strains with different deletions within the capsule biosynthesis cluster have been reported to produce less biofilm, not more [21,22,40]. It is not known, however, how expression levels of the *pga* operon might have changed in these other mutants. In addition, type 1 fimbriae have been reported to play an important role in biofilm formation in a non-encapsulated *K. pneumoniae* mutant [23], but no fimbriae was necessary for increased biofilm formation in the non-encapsulated mutant we generated (Figure 3). Taken together, these observations suggest that overlapping regulatory mechanisms likely act to regulate these three features in *K. pneumoniae* in a strain-specific manner.

## Competing interests

The authors declare that they have no competing interests.

## Authors’ contributions

TWH, IL, and PC carried out the experiments. TWH, HYC, SFT, BOP, and PC designed the study. All authors participated in manuscript preparation. All authors have read and approved the final manuscript.

## Supplementary Material

Additional file 1 All primers used in this study for plasmid construction and to generate and confirm the knockouts. Click here for file

Additional file 2 **Overview of the λ-Red based protocol to create gene knockouts in *Klebsiella pneumoniae.***
Click here for file

Additional file 3 **The fold change of *pga*** **ABC and *mrk* operons in Δwzabc and Δcps mutants.** Click here for file
