# Supplementary material for: Capsule deletion via a λ-Red knockout system perturbs biofilm formation and fimbriae expression in Klebsiella pneumoniae MGH 78578
Source: BMC Res Notes. 2014 Jan 8;7:13. doi: 10.1186/1756-0500-7-13 (PMC3892127; doi:10.1186/1756-0500-7-13)
Supplement: Additional file 1 — All primers used in this study for plasmid construction and to generate and confirm the knockouts. [file 1756-0500-7-13-S1.docx]

**Additional file 1.** Primers used in this study

| Primer name | Sequences |
| --- | --- |
| Primers for cloning |  |
| CT-bla-F | CTGTCAGACCAAGTTTACTCATATATA |
| CT-bla-R | CATACTCTTCCTTTTTCAATATTATTG |
| Hyg-F | /5’ Phosph/AAAAAGCCTGAACTCACCGC |
| Hyg-R | /5’ Phosph/CTATTCCTTTGCCCTCGGAC |
| BSR-fwd-Xh^a^ | ATAC**CTCGAG**TTTTTTTAAGGCAGTTATTGGTGC |
| BSR-rev-Bg^a^ | CCCG**AGATCT**GCAAAGTGCGTCGGGTGATG |
| BlaHyg-fwd-Bg^a^ | TTGC**AGATCT**CGGGGAAATGTGCGCGGAAC |
| BlaHyg-rev-Xh^a^ | AAAA**CTCGAG**GTATATATGAGTAAACTTGGTCTG |
| p15A-5’ | GTGCACCGATGATAAGCTGT |
| Hyg-R-1 | CTATTCCTTTGCCCTCGGAC |
|  |  |
| Primers for knock out | |
| wza-FRT19^b^ | AGGAATTGGCAAATTTTGATTTACTGATGATGTGACATTTGTAGGCTGGAGCTGCTTC |
| wzc-FRT20^b^ | CCGTCCTTTTAGTTTATTAAATTTTATCGGTTTTCCGAAATTCCGGGGATCCGTCGACC |
| cps_KO_Fwd^b^ | GATTTGTGACATGTACAATTTTGCAAAACCGCGTGTTTTGCAACCAAACAGGTGAAGATGATTCCGGGGATCCGTCGACC |
| cps_KO_Rev^b^ | GGGGGAGAGAAAGAAGAACACTGGCGGCAGGGAACCGCCAGTGTCAGACAGGCAGAATTATGTAGGCTGGAGCTGCTTC |
|  | |
| Primers for verification^c^ | |
| Scar-Fwd | CTAGAGAATAGGAACTTCGAAC |
| wzc-down-5' | GGATCATGTGCCAGAACAGG |
| wzc-Rev | GAGGTCGGTATTAAACGCTTCG |
| wza-up-3' | GTGAGTTTCTATGGGCAAATG |
| cps_Conf_Fwd | GGATTCAGGCTAATATGTCG |
| cps_Conf_Rev | GCGTTTTGGCATGGCCTTAA |
| galF-Rev | GCTCGTAGGAGGTATCGAAG |
|  | |
| Primers for plasmids of MGH 78578 | |
| PifA-5' (pKPN5) | CCAGGTCCGGGATGACAC |
| PifA-3' (pKPN5) | GCTTGTCGCAGACGGTATG |
| MerC-5' (pKPN4) | CCAGTTCCTGCACCGTCATC |
| MerC-3' (pKPN4) | CGGCACCTGCGTCAATGTC |
| ArsA-5' (pKPN3) | CAATATCGGGCCAGAATCG |
| ArsA-3' (pKPN3) | CTCATCAACTCTCTGAGCTTT |

**^a^** Bold letters mean the extra-added restriction enzyme cutting site

**^b^** The underline means the annealing sites to DNA templates during PCR amplification.

**^c^** In Fig. 2B of the main text, the composition of each primer pair is (1) wzc-down-5’ and wzc-Rev; (2) wzc-down-5’ and Scar-Fwd; (3) cps_Conf_Fwd and galF-Rev; (4) cps_Conf_Fwd and Scar-Fwd.
