# Supplementary material for: Capsule deletion via a λ-Red knockout system perturbs biofilm formation and fimbriae expression in Klebsiella pneumoniae MGH 78578
Source: BMC Res Notes. 2014 Jan 8;7:13. doi: 10.1186/1756-0500-7-13 (PMC3892127; doi:10.1186/1756-0500-7-13)
Supplement: Additional file 2 — Overview of the λ-Red based protocol to create gene knockouts in Klebsiella pneumoniae. [file 1756-0500-7-13-S2.docx]

**Additional file 2: Overview of the λ-Red based protocol to create gene knockouts in *Klebsiella pneumoniae.***

Three plasmids are required in our procedure. Plasmid pIJ773 served as the template to amplify the apramycin resistance gene, *aac(3)IV*, and flanking FRT sites [[33](#_ENREF_33)]. Plasmid pACBSR-Hyg contains the same λ-Red system that is used to construct knockouts in *E. coli* and is used to facilitate homologous recombination between the knockout cassette and the target locus in the chromosome. The three genes that comprise this system, *beta, gam,* and *exo*, are under the control of an arabinose-inducible promoter. This plasmid has a temperature-sensitive replication origin; it replicates at 30 °C but not 37 °C. Plasmid pFLP-Hyg contains the FLP recombinase, which was used to excise the apramycin selection marker from the chromosome via the FRT sites. The selection marker for both pACBSR-Hyg and pFLP-Hyg is hygromycin resistance.

To construct the knockouts, we first introduced plasmid pACBSR-Hyg into wild-type *K. pneumoniae* through electroporation. One milliliter of an overnight culture was inoculated into 100 ml LB broth and placed in a 30 °C incubator. A magnetic stir bar inside the flask spinning at approximately 500 rpm was used for aeration. When the culture reached an optical density at 600 nm (OD_600_) of 0.6 to 0.8 (approximately four hours), the cells were centrifuged at 6500 rpm for five minutes and washed twice with 50 ml ice-cold 10% glycerol. Cells were resuspended in the residual glycerol solution after the final wash. A 50 μl aliquot of the dense suspension was then mixed with 200 to 400 ng of plasmid DNA and electroporated at 2500 mV. SOC medium was added immediately after electroporation. The sample was then incubated for one hour at 30 °C on a rotary platform shaker at 150 rpm and then spread onto low salt LB plates containing hygromycin.

The knockout cassette consisted of three distinct regions: the *aac(3)IV* gene that confers apramycin resistance, FRT sites that flanked *aac(3)IV*, and 60 bp regions at each end that were homologous to the chromosomal region to be deleted. We constructed this cassette by ordering PCR primers that contained the 60 bp homologous regions on the 5’ end of each primer and consensus sites used to amplify *aac(3)IV* and the flanking FRT sites from plasmid pIJ773 on the 3’ ends [[33](#_ENREF_33)]. PCR amplification using pIJ773 as the template was then used to make the cassette.

The deletion mutant was constructed through allelic exchange between the knockout cassette and the chromosomal region of interest. Specifically, the cassette was electroporated into *K. pneumoniae* bearing pACBSR-Hyg as described above, but with the following modifications: 1) instead of standard LB, the culture broth consisted of 90 ml low salt LB, 10 ml of a sterile 1 M L-arabinose solution, and 100 μg/ml hygromycin; 2) 500 to 1000 ng of the knockout cassette was used for electroporation; and 3) after transformation, cells were plated on LB agar plates containing apramycin and incubated overnight at 37 °C. The next day, we performed PCR to screen for colonies that had a correct insertion of the knockout cassette. Similar to the *E. coli* knockout protocol, we used four sets of primers for screening: one that spanned the full length of the knockout cassette; a second that targeted the 5’ junction; a third that targeted the 3’ junction; and a fourth that amplified *aac(3)IV* only. After we identified colonies that had the correct insertion, they were serially passed on LB plates containing apramycin for several days until they became sensitive to hygromycin again, indicating loss of pACBSR-Hyg.

The next step in the procedure is to excise the knockout cassette. Colonies that had the correct insertion and had lost pACBSR-Hyg were transformed with 200 to 400 ng of pFLP-Hyg plasmid DNA using the electroporation protocol described above. The selective medium for this step was low-salt LB plates with hygromycin. After overnight incubation at 30 °C, 16 to 20 colonies were streaked onto an LB plate and heat-shocked at 43 °C overnight. This step induces the FLP recombinase, which facilitates excision of *aac(3)IV* through homologous recombination between the two flanking FRT sites. The plate was removed from the 43 °C incubator the next day, and all replicates were streaked onto matching LB and LB + apramycin plates to screen for loss of *aac(3)IV*. Replicates that had regained apramycin sensitivity were serially passed on LB plates at 37 °C until they had also regained hygromycin sensitivity, indicating loss of pFLP-Hyg. This process took approximately five to ten days. Lastly, we extracted DNA from colonies that had become sensitive to both apramycin and hygromycin and sequenced the affected chromosomal region to confirm the knockout. This procedure leaves behind an 81 bp scar region.

The full knockout protocol and the necessary plasmids are available upon request.

**
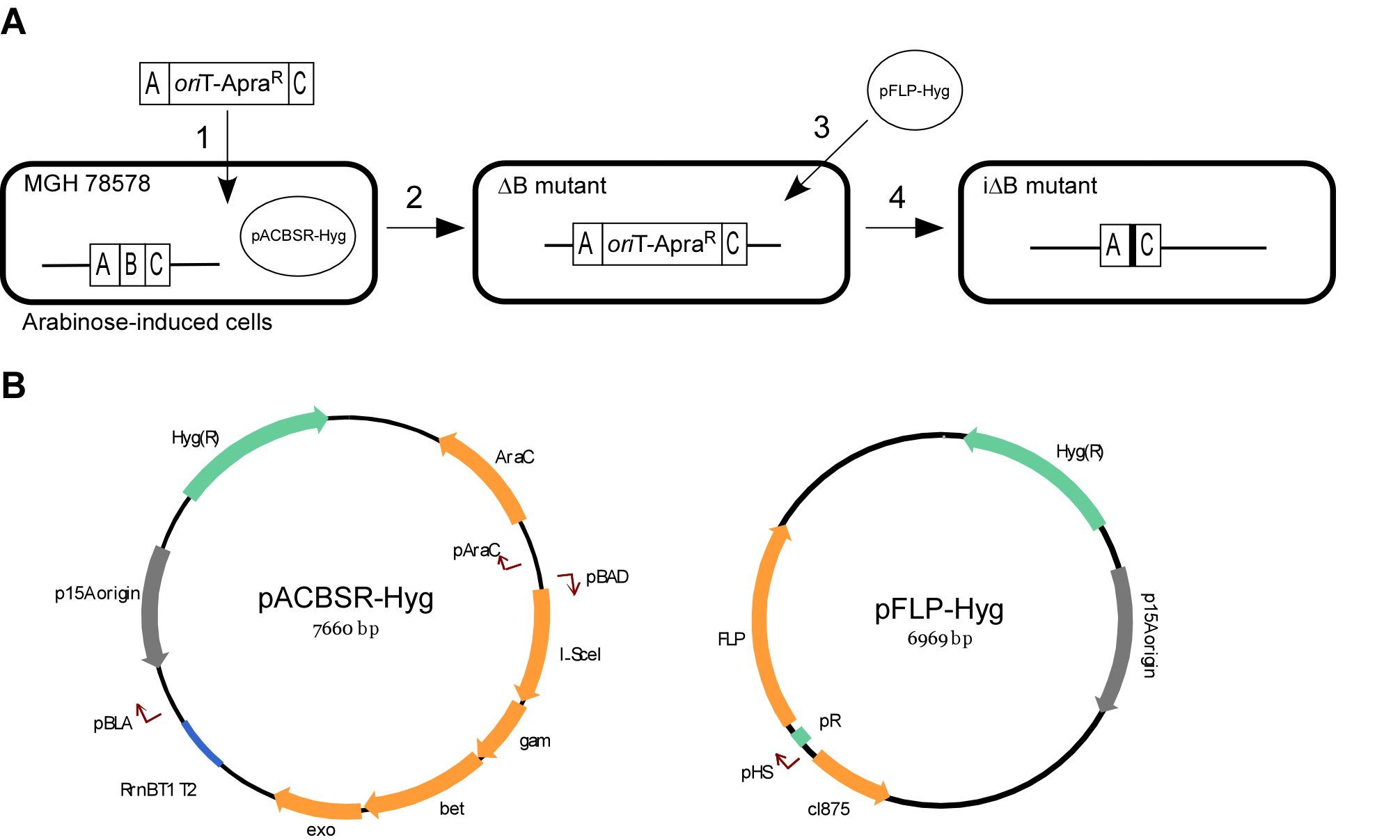
**

**A.** Overview of the λ-Red-based knockout procedure developed here to create gene knockouts in *K. pneumoniae*. The locus to be deleted is denoted by the letter B. The four major steps are: 1) transform a knockout cassette bearing the *aac(3)IV* gene flanked by ~60 bp sequences immediately upstream and downstream of the region to be deleted into cells expressing Red recombinase off of pACBSR-Hyg; 2) select for apramycin-resistant transformants and loss of pACBSR-Hyg; 3) transform with pFLP-Hyg; and 4) induce the FLP recombinase and screen for loss of apramycin resistance. The *aac(3)IV* marker, which confers apramycin resistance, can now be reused to create additional deletions. **B.** Plasmid maps for pACBSR-Hyg and pFLP-Hyg. The I-SceI locus on pACBSR-Hyg is a remnant that is not used in this protocol.
