# Supplementary material for: Capsule deletion via a λ-Red knockout system perturbs biofilm formation and fimbriae expression in Klebsiella pneumoniae MGH 78578
Source: BMC Res Notes. 2014 Jan 8;7:13. doi: 10.1186/1756-0500-7-13 (PMC3892127; doi:10.1186/1756-0500-7-13)
Supplement: Additional file 3 — The fold change of pga ABC and mrk operons in Δwzabc and Δcps mutants. [file 1756-0500-7-13-S3.docx]

**Additional file 3.** The fold change of *pga*ABC and *mrk* operons in Δwzabc and Δcps mutants.

| Locus_tag | Gene name | Expression level | | |  | Δwzabc | |  | Δcps | |
| --- | --- | --- | --- | --- | --- | --- | --- | --- | --- | --- |
|  |  | WT | iΔwzabc | iΔcps |  | T-test | Fold |  | T-test | Fold |
| KPN_03273 | *mrkI* | 12.23 | 11.88 | 11.1 |  | 0.0927 | 0.79 |  | 0.0065 | 0.45 |
| KPN_03274 | *mrkJ* | 10.43 | 9.63 | 9.12 |  | 0.0112 | 0.58 |  | 0.0157 | 0.40 |
| KPN_03275 | *mrkF* | 12.52 | 12.54 | 11.1 |  | 0.7694 | 1.01 |  | 0.0048 | 0.38 |
| KPN_03276 | *mrkD* | 13.46 | 13.17 | 11.9 |  | 0.0036 | 0.82 |  | 0.0007 | 0.35 |
| KPN_03277 | *mrkC* | 13.31 | 12.89 | 11.7 |  | 0.0632 | 0.75 |  | 0.0000 | 0.33 |
| KPN_03278 | *mrkB* | 13.90 | 13.49 | 12.3 |  | 0.1155 | 0.75 |  | 0.0012 | 0.32 |
| KPN_03279 | *mrkA* | 15.52 | 15.53 | 15.2 |  | 0.8945 | 1.00 |  | 0.1208 | 0.81 |
|  |  |  |  |  |  |  |  |  |  |  |
| KPN_03285 | *fimB* | 9.19 | 9.32 | 8.99 |  | 0.0496 | 1.10 |  | 0.1788 | 0.87 |
| KPN_03286 | *fimE* | 7.63 | 8.00 | 8.04 |  | 0.0287 | 1.30 |  | 0.0092 | 1.34 |
| KPN_03287 | *fimA* | 12.66 | 12.54 | 11.20 |  | 0.5990 | 0.92 |  | 0.0276 | 0.36 |
| KPN_03288 | *fimI* | 9.63 | 9.19 | 8.37 |  | 0.0472 | 0.74 |  | 0.0120 | 0.42 |
| KPN_03289 | *fimC* | 8.89 | 8.46 | 7.79 |  | 0.1242 | 0.74 |  | 0.0320 | 0.47 |
| KPN_03290 | *fimD* | 7.44 | 7.13 | 6.86 |  | 0.1172 | 0.81 |  | 0.0733 | 0.67 |
| KPN_03291 | *fimF* | 7.22 | 7.16 | 6.84 |  | 0.6726 | 0.96 |  | 0.1069 | 0.77 |
| KPN_03292 | *fimG* | 7.53 | 7.34 | 6.95 |  | 0.0869 | 0.88 |  | 0.0549 | 0.67 |
| KPN_03293 | *fimH* | 7.73 | 7.61 | 7.31 |  | 0.4039 | 0.92 |  | 0.0598 | 0.75 |
| KPN_03294 |  | 6.91 | 6.81 | 6.72 |  | 0.3686 | 0.93 |  | 0.1233 | 0.88 |
|  |  |  |  |  |  |  |  |  |  |  |
| KPN_04512 | *pgaC* | 6.59 | 7.10 | 9.93 |  | 0.0221 | 1.43 |  | 0.0006 | 10.09 |
| KPN_04513 | *pgaB* | 6.57 | 7.10 | 9.98 |  | 0.0102 | 1.45 |  | 0.0003 | 10.64 |
| KPN_04514 | *pgaA* | 6.69 | 7.73 | 11.13 |  | 0.0014 | 2.06 |  | 0.0001 | 21.68 |
| KPN_04515 |  | 6.79 | 8.47 | 12.50 |  | 0.0005 | 3.21 |  | 0.0031 | 52.11 |
| KPN_04516 |  | 7.85 | 8.36 | 11.37 |  | 0.0191 | 1.42 |  | 0.0006 | 11.52 |
